# Meetings of Societies

**Published:** 1934

**Authors:** 


### Meetings of Societies

### Bristol Medico-Chirurgical Society

THE fourth meeting of the Society was held in the Physiological Lecture Theatre of the University on Wednesday, 10th January, at 8.30 p.m.

Dr. Herbert Rogers showed and explained pathological specimens, including one from a case of ulcerative endocarditis, and a short discussion followed, in which Mr. Wright, Dr. P. Watson-Williams and Dr. Perry joined, and Dr. Rogers replied.

The minutes of the preceding meeting were read and confirmed.

The President announced that the Dinner Committee had arranged that the annual dinner of the Society would be held at the Berkeley Café on Wednesday, 7th March.

Mr. E. Watson-Williams then read a paper on **"Catarrh,"** illustrating his paper with cases and the epidiascope. The President thanked and congratulated Mr. Watson-Williams, and in the discussion which followed Mr. Wright, Dr. P. Watson-Williams, Professor Nixon, Mr. Angell James, Dr. Herbert Rogers and Dr. Alexander took part, and Mr. E. Watson-Williams replied.

---

THE fifth meeting of the session was held in the Physiological Lecture Theatre of the University on Wednesday, 14th February, at 8.30 p.m., Dr. Elwin Harris (sen.) presiding, and 118 members and visitors being present.

Dr. G. B. Bush and Dr. A. L. Taylor showed skiagrams and pathological specimens respectively.

The minutes of the preceding meeting were read and confirmed.

The President then spoke of the death of Dr. W. P. Kennedy, and at his request the members stood in silence for a few moments.

Dr. A. L. Taylor, at the request of the President, described the pathological specimens which he had shown.

The President then introduced Sir Thomas Peel Dunhill, who gave an address on **"Toxic Goitre."**

The President thanked and congratulated Sir Thomas Dunhill, and questions were asked by Mr. Clifford Moore, Dr. H. H. Carleton, Professor Nixon, Mr. Walters, Dr. P. Watson-Williams, Dr. Frank Bodman, and Dr. Sutton, to which Sir Thomas Dunhill replied.

Professor Rendle Short proposed and Mr. C. A. Moore seconded a vote of thanks to Sir Thomas Dunhill for his address, and this was carried with acclamation.

